# Esthetic and Physical Changes of Innovative Titanium Surface Properties Obtained with Laser Technology

**DOI:** 10.3390/ma13051066

**Published:** 2020-02-28

**Authors:** Filiberto Mastrangelo, Raimondo Quaresima, Roberto Abundo, Gianrico Spagnuolo, Gaetano Marenzi

**Affiliations:** 1 Department of Clinical and Experimental Medicine, University of Foggia, 71122 Foggia, Italy; 2 Department of Civil Engineering, Architecture and Environment, University of L’Aquila, 67100 L’Aquila, Italy; raimondo.quaresima@univaq.it; 3 Private Practice, 10121 Turin, Italy; robabund@yahoo.it; 4 Department of Neurosciences, Reproductive and Odontostomatological Sciences, Federico II University, 80100 Naples, Italy; gspagnuo@unina.it (G.S.); gaetano.marenzi@gmail.com (G.M.)

**Keywords:** titanium surfaces, laser treatment, titanium colors, dental implant, osseointegration, periodontal integration

## Abstract

Aim: The purpose of the study was the evaluation of the esthetic and physical changes produced on colored titanium Grade 5 (Ti6Al4V) laser treated surfaces to be used in implant dentistry for esthetic success. Materials and methods: Colored titanium surfaces were obtained with laser treatment. The physical and topographic properties were evaluated by stereo, light, and electron microscopy and profilometric analyses. L*a*b* colorimetric coordinates were measured by spectrometry, and the superficial chemical characteristics were evaluated by energy dispersive X-ray analysis. Results: Within the complete palette of titanium colors, pinks (P1-P2), incarnadine (I), and white (W) obtained by laser were selected. The topography, texture, hues, saturation, roughness, and porosity of the samples were compared with those of machined (M) and sand-blasted and etched (SBAE) control surfaces. P1, P2, and I, similar in hue and roughness (Ra ≅ 0.5 μm), had a microgroove spacing of 56 μm and a decreasing porosity. The W sample with a “checkerboard” texture and a light color (L* 96.31) was similar to the M samples (Ra = 0.32 μm), but different from SBAE (Ra = 1.41 μm, L* 65.47). Discussion: The aspects of hard and soft tissue could result in an esthetic failure of the dental implant by showing the dark color of the fixture or abutment. The two different pinks and incarnadine surfaces showed favorable esthetic and physical features to promote dental implant success even in the maxillary anterior area with gingival recession, asymmetry, and deficiency. Conclusion: Titanium colored laser surfaces represent a valid alternative to those currently traditionally obtained and interesting and potential perspectives in the management of dental implants’ esthetic failure.

## 1. Introduction

In 2016, the World Health Organization (W.H.O.) considered complete or partial edentulism to be one of the leading ten causes of Years Lived with Disability [1], and in 2018, the American Dental Association defined osseointegrated dental implants as a valid and predictable therapy for the rehabilitation of the jaws with a high success rate (<90%) [2,3]. In 1986, Albrektson defined the success criteria of dental implants as a clinical condition with the absence of fixture mobility and pain, the absence of radiographic radiolucency, the absence of infection, and minimal bone loss (<1 mm/y) [4,5]. In 1999, Askary introduced the concept of functional, esthetic, or phonetic criteria to define dental implant failure [6]. Currently, the success parameters have evolved, including the soft tissue response and the aesthetic parameters, as the crucial criteria for the success of implant dentistry [7,8]. Today, the focus of patients is shifting from implant function to the esthetic restoration value to combine osseointegration success with peri-implant natural teeth and soft tissue evaluation [9,10], making maxillary anterior dentistry the highest challenge of modern implantology according to the Straightforward, Advanced, and Complex International Team for Implantology (ITI) classification [11]. Therefore, esthetic failure has become particularly important since 2009, and a wide literature and different guidelines have been produced. Bone loss and soft tissue damage can determine the esthetic failure of the implant with colors like gray or silver that should not be present in the oral cavity, as the gingival and dental tissues’ colors are pink and white [12]. In the early 19^th^ Century, Nobili investigated metal oxidation to produce different nanometer thick oxide films capable of generating chromatic effects through the interaction with natural or artificial light radiation [13,14]. Many studies showed the physical principles underlying the titanium color’s formation through oxide thickness combined with the anodization procedures [15,16], ensuring a smooth titanium surface with brilliant hues that are reproducible and durable [17], but do not favor the implant osseointegration [18,19] and periodontal tissue integration process, which instead require titanium surfaces with micro- and nano-roughness [20,21].

The aim of the present in vitro study was to realize innovative titanium colored surfaces with a laser subtractive technology compared to the current traditional ones. The second purpose was to evaluate and discuss if their esthetic and physical features could be suitable to be used in implant dentistry.

## 2. Materials and Methods 

Five specimens of titanium Grade 5 (Ti6Al4V) (Titanium Alloy-EuropaAcciai, Chieti, Italy) (140 × 140 × 0.5 mm^3^), mechanically obtained by cutting from a machined plate, were ultrasonically cleaned in acetone (Elma Elmasonic S 60/H, Germany—RPE Carlo Erba, Italy) for 10 min, then in Millipore water for the same time, and dried in a thermostatic oven (20 °C for 3 h). With specific different selected sequences of nano-second pulses, in a cleaning chamber, the specimens were ablated using an ytterbium laser. Among all the titanium colors obtained (palette) (12 × 12 mm^2^ only Pink Type 1 (P1), Pink Type 2 (P2), incarnadine (I), and white (W) colors were selected for the study. Samples of machined (M) and sandblasted and acid etched (SBAE) titanium surfaces were used as the control.

### Surface Analyses

At the Microscopy Center of the University of L’Aquila, the topographic features of all samples were evaluated by stereo-microscopy (M125C, Z6APOA, Leica, Canon Power Shot 650 IS, Ohta-ku, Tokyo, Japan), optical microscopy (Nikon Optiphot2, Tokyo, Japan), and electron microscopy (Philips XL30CP, AE Eindhoven, The Netherlands). Chemical surface contamination was assessed by energy dispersive spectroscopy (Oxford, Inca Energy 250, High Wycombe, UK) at a voltage of 20 kV; spectra were collected at a magnification of 1000× and a collection time of 1 min, and the formation of titanium compounds was evaluated by X-ray diffraction (XRD) (Philips PW 1729, AE Eindhoven, The Netherlands) using CuKα radiation with a step size of 0.02° 2ϑ in the 5°–90° 2ϑ range and operating with a 1st divergence slit at 30 kV/40 mA. The crystalline phases were detected using the Joint Committee of Powder Diffraction Standard database (JPDS) of the International Centre for Diffraction Data [13].

The color analysis of the samples was performed at the Civil Engineering, Architecture and Environment Department of L’Aquila University by spectrophotometry (X-Rite SP64) with a diffuse illumination integrating sphere system (illuminant and reference angle for the output values of D50 2°).

The values obtained belonged to the colorimetric space CIELAB, which is defined as a standard colorimetric space by the International Commission on Illumination. It expresses color as three values: L* for lightness from black (0) to white (100), a* from green (−) to red (+), and b*from blue (−) to yellow (+). In the CIELAB system, the same amount of numerical change in the Lab values corresponds to roughly the same amount of visually perceived change. The CIELAB system allows having a measure of the color that approximates human subjective vision (L* = 0 yields black and L* = 100 indicates diffuse white; specular white may be higher); values between red/magenta and green are defined by a* (negative values indicate green, while positive values indicate magenta), while values between yellow and blue by b* (negative values indicate blue and positive values yellow) [13].

The color variations between controls and colored surfaces were detected by the CIELAB recommendations as $\Delta E_{ab}^{*}=\sqrt{{\left(L_{2}^{*}-L_{1}^{*}\right)}^{2}+{\left(a_{2}^{*}-a_{1}^{*}\right)}^{2}+{\left(b_{2}^{*}-b_{1}^{*}\right)}^{2}}$ [13].

Profilometric values were assessed at the Industrial and Information Engineering and Economics Department of L’Aquila University by a profilometer (Taylor Hobson, Subsonic 3+, Leicester, UK). All specimens’ Ra (average linear surface roughness), Rq (surface root mean squared), Rz (surface average distance between the highest peak and lowest valley), Ry (maximum peak height), and Sm (mean spacing between peaks) measurements of a 4 mm area (point density of 500/mm), along the orthogonal and diagonal baselines were performed in triplicate. 

## 3. Results

**Figure 1:**
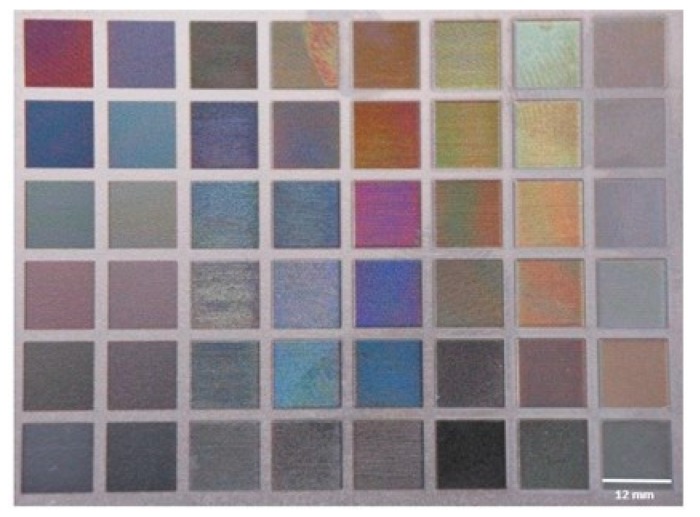
Palette of titanium Grade 5 surface with the sixty-four colors obtained by laser ablation (the dimensions of each single colored field are 12 × 12 mm^2^).

**Figure 2:**
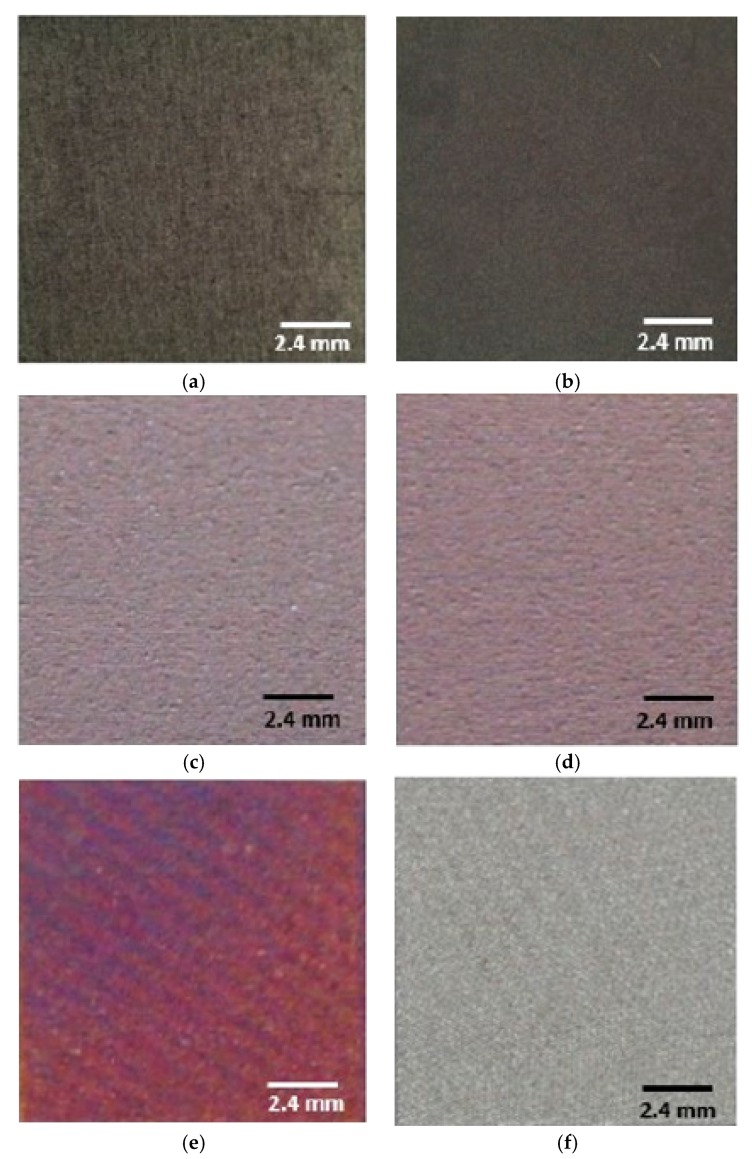
Stereomicroscope analysis of the chosen titanium samples: (**a**) machined (M) (**b**) sand blasted and acid etched (SBAE), (**c**) Pink Type 1 (P1), (**d**) Pink Type 2 (P2), (**e**) incarnadine (I), and (**f**) white (W).

**Figure 3:**
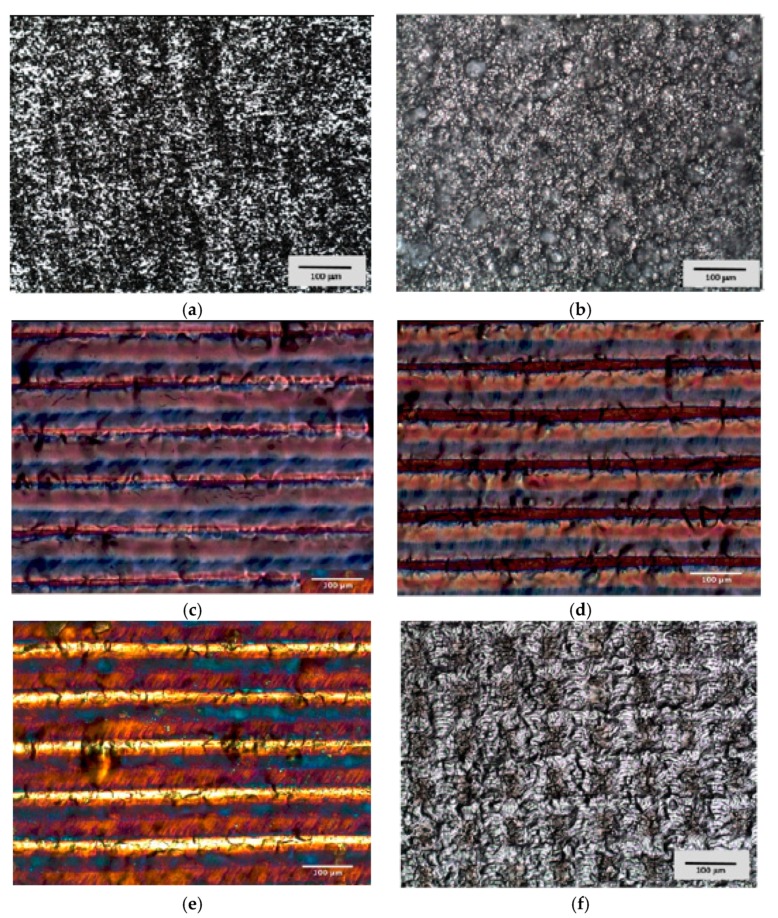
Light microscopy analysis of (**a**) M, (**b**) SBAE, (**c**) P1, (**d**) P2, (**e**) I, and (**f**) W samples (400×). A typical metallic aspect and a high random roughness were detected for both control surfaces, while the laser treatment produced a texture of parallel patterns (microgrooves) in P1, P2, and I. In the W sample, the laser treatment generated a fine perpendicular pattern having only a few color components (grey scale).

The complete palette of titanium colors from the stereo microscope analysis, in Figure 1, is reported; in particular, it is possible to note (Figure 2) the typical metallic aspect in M (a) samples with a bright lightness, a lower reflectance in SBAE samples (b), while higher lightness, iridescence, and interference phenomena of P1 (c), P2 (d), I (e), and W (f) colored surfaces. Different topographic aspects of the specimens were observed with light microscopy analyses. (Figure 3). 

In laser samples, it was possible to observe a different parallel micro-grooved texture deeper in P1 than P2 and I (Figure 3c–e) The W specimens showed a complex texture with perpendicular microgrooves. (Figure 3f) The control surfaces showed lower uniform and parallel rolling lines in M samples, and a random texture with different peaks and valleys in SBAE. 

**Figure 4:**
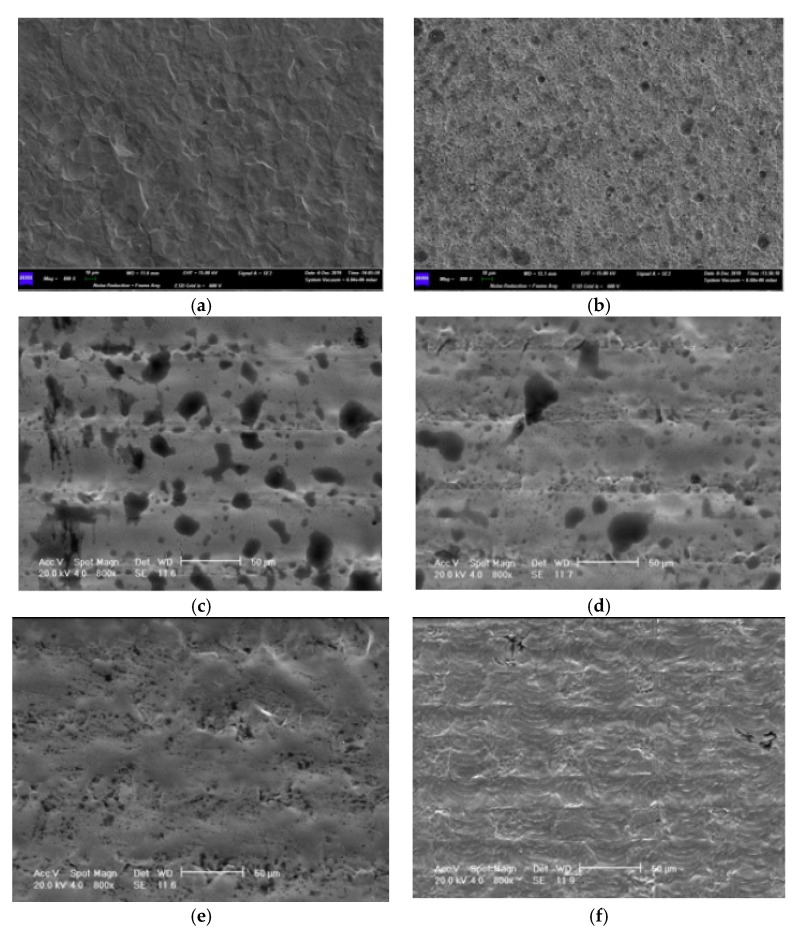
High magnification (800×) SEM analysis of (**a**) M, (**b**) SBAE, (**c**) P1, (**d**) P2, (**e**) I, and (**f**) W surfaces. Different topographic features and roughness characteristics were observed. The M sample showed a smooth surface, while a high random roughness with pits in SBAE specimens was observed. P1 and P2 samples showed a smooth surface with pores and evident microgrooves. For the I surface, smoother lines with a smaller pore size distribution were observed. The W surface showed smoother lines compared with the M samples even if it had a “checkerboard” texture made of perpendicular grooves. The porosity (quantity and dimension of the pores) decreased gradually from high to low values for the P1, P2, I, and W laser surfaces.

The electron microscopy analysis (SEM) (800×) of P1 and P2 showed similar parallel groove spacings (56 μm) (Figure 4). The P1 specimens revealed a high pit porosity, ranging between 2 and 31 μm, with an irregular distribution more predominant between 12 and 31 μm (Figure 4c). The P2 pit porosity showed a lower value (range 2–31 μm) with a distribution between 6 and 8 μm (Figure 4d). In I samples, a 2–4 μm pit porosity was observed, while the microgroove spacing value was not detectable (Figure 4e). The W samples showed a 28 μm spacing value with the lowest porosity of 12 μm. (Figure 4f). The M samples showed a smooth surface with the absence of pits and valleys (Figure 4a). In SBAE controls, it is possible to appreciate the random distribution of the pits (range 1–15 μm) and the highest different roughness topography (Figure 4b).

**Figure 5:**
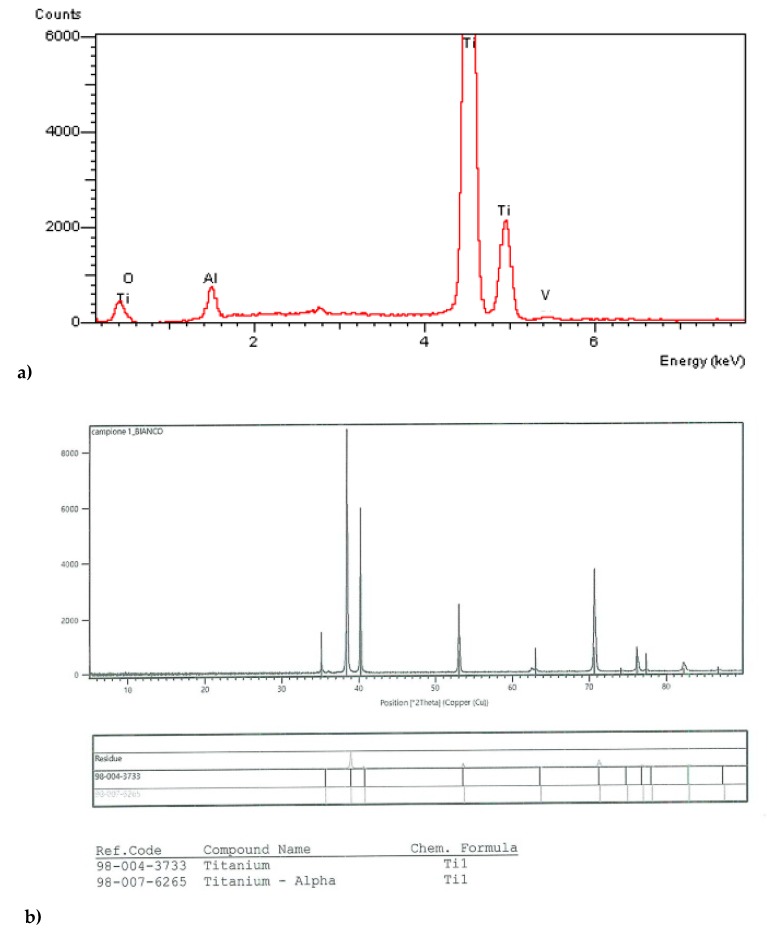
Laser ablated colored surfaces of P1, P2, I, and W: (**a**) Energy dispersive spectroscopy analysis and (**b**) X-ray diffraction; the absence of impurities and foreign titanium compounds are shown, respectively.

On all the surfaces of the P1, P2, I, and W samples, the absence of chemical contamination was assessed (Figure 5a,b).

According to CIE L*a*b*, the P1, P2, and I color analysis showed the same hue with different saturation values, while the W samples had completely different hues and no comparable values. The L* 88.42, a* 45.34, b* 39.21 color coordinates with 64.30 ΔE in the P1 specimens were detected. The P2 samples showed L* 90.65, a* 43.72, b* 37.31, ΔE 62.75. The I samples showed L* 34.27, a* 44.53, b* −21.92, and 59.80 ΔE, and W samples L* 96.31, a* 2.45, b* −10.91, ΔE 30.69. The M color coordinates were 67.64, −0.39, −0.99, in SBAE 65.47, −0.49, −0.99. The SBAE ΔE value was 2.17, while the M value was zero (Table 1).

The profilometric analysis showed similar surface roughness values for P1, P2, and I, while the W and M samples’ values were analogous. The SBAE analysis detected a higher value of surface roughness in all parameters evaluated. P1 showed Ra 0.44 μm - Rq 0.58 - Rz 3.24 μm- Ry 1.93 - Sm 54 μm values. In P2 samples, the Ra was 0.54 μm, Rq 0.72 - Rz 3.86 - Ry 2.61, and Sm 66 μm. The I specimens showed Ra 0.52 μm - Rq 0.67 - Rz 3.47 - Ry 2.14 - Sm 66 μm and the W sample Ra 0.32 μm - Rq 0.41 - Rz 2.21 - Ry 1.21 - Sm 37 μm values. For M surfaces Ra 0.32 μm, Rq 0.47 - Rz 2.21 μm Ry 2.74, Sm 53 μm were observed, while for the SBAE surfaces, Ra 1.41 μm, Rq 21.92 - Rz 12.87, Ry 2.74, and Sm of 83 μm were detected (Table 2).

## 4. Discussion

After the ITI conferences, modern dental implantology defined the esthetic success of the maxillary anterior area as the highest challenge including gingival and dental tissues’ response around the implants [11]. Bone loss, thin gingiva biotype, high smile lines, and adverse esthetic affects could result in an esthetic failure showing the dark color of the conventional titanium dental implant fixture or abutment [22]. Several chemical, thermal, and electrochemical methods are used to change the titanium surfaces’ properties by means of the formation of a TiO2 film in order to achieve osseointegration properties [23]. All the methods improve the esthetic colored coatings or layers, but chemical treatment might produce allergic reactions, while thermic methods produce a uniform and non-reproducible color with low corrosion resistance [24,25,26]. Recent studies showed that the anodic oxidation procedures could promote the color formation of the abutments in response to the gingival esthetic features [17]. The anodic treatment produces various colors with improved corrosion resistance through the increased thickness of the oxide layer [27]. However, the anodic treatment produces significant environment and working risks and a typical, smooth surface [28] unfavorable to the osseointegration process [29,30]. The alternative and innovative titanium laser treatment produced a complete palette of colors with different optical and physical features. The optical and stereomicroscope evaluation showed that various colors of the titanium surface could be produced with higher lightness, iridescence, and light interference with the oxide layer more similar to the oral soft tissues’ optical characteristics. Laser treatment showed several optical advantages as lower hue and saturation features, different and reproducible roughness textures, and lower iridescence, probably due to a re-solidification of the titanium after the laser ablation. According to CIE, the color evaluation showed hue, saturation, and ΔE values more similar to the soft tissue [22]. The two different laser obtained pinks and incarnadine surfaces are suitable to be used for esthetic success in gingival recession and asymmetry, papillary deficiency, and gingival tissue graying. The laser obtained white color showed optical features not completely adequate for enamel, ceramic, or zirconia and more similar to machined titanium. Different spaces of the laser obtained microgrooves showed similar profilometric values and scanning microscopy measurements capable of promoting cells’ alignment and migration [25,26,30,31,32]. Compared with the traditional titanium surface treatment, the laser ablation method can be considered particularly efficient, faster, highly reproducible, environmentally suitable, and economic. Furthermore, the laser treatment simplifies the procedure and reduces implant fixture manufacturing time, showing a cleaned and sterilized surface with the absence of chemical contaminants.

## 5. Conclusions

Further studies will be needed to validate the tissue response to the laser obtained colored titanium surfaces. However, these preliminary results showed how the laser technology produced innovative physical titanium changes with a wide range of hues and saturations of vivid colors. The light interference with the oxide titanium layer improved the optical color properties with potential biological and clinical applications in implant dentistry. The roughness of the titanium laser ablated surfaces should promote the osseointegration process. The absence of impurities, the sterilized surface, and the reproducible, eco-friendly, and fast manufacturing confirmed the laser technology as a useful tool to produce a large number of implant fixtures and abutments with a substantial reduction of the time and cost, and it appears to be a valid alternative to traditional titanium surface treatment. 

## Figures and Tables

**Table 1:** Chromatic coordinates, expressed in the L*a*b* system, of surfaces machined (M), sand blasted and acid etched (SBAE), Pink Type 1 (P1), Pink Type 2 (P2), incarnadine (I), white (W), and color variation (ΔE) compared with the machined titanium surface.

Sample	L*	a*	b*	ΔE
**M**	67.64	−0.39	−0.99	-
**SBAE**	65.47	−0.49	−0.89	2.17
**P1**	88.42	+45.34	+39.21	64.30
**P2**	90.65	+43.72	+37.31	62.75
**I**	34.27	+44.53	−21.92	59.80
**W**	96.31	+2.45	−10.91	30.69

**Table 2:** Roughness parameters, reported in microns, of machined (M), sand blasted and acid etched (SBAE), Pink Type 1 (P1), Pink Type 2 (P2), incarnadine (I), and white (W) titanium surfaces.

Sample	Ra	Rq	Rz	Ry	Sm
M	0.32 (±0.02)	0.47 (±0.03)	2.21 (±0.19)	2.74 (±0.15)	53 (±4.86)
SBAE	1.41 (±0.19)	21.92 ±1.31)	12.87 (±1.39)	2.52 (±0.13)	83 (±8.73)
P1	0.44 (±0.02)	0.58 (±0.03)	3.24 (±0.27)	1.93 (±0.10)	54 (±4.99)
P2	0.54 (±0.03)	0.72 (±0.05)	3.86 (±0.36)	2.61 (±0.14)	66 (±6.96)
I	0.52 (±0.03)	0.67 (±0.04)	3.47 (±0.31)	2.14 (±0.12)	66 (±6. 31)
W	0.32 (±0.02)	0.41 (±0.02)	2.21 (±0.18)	1.21 (±0.12)	37 (±4.01)
